# Supplementary material for: Soybean–SCN Battle: Novel Insight into Soybean’s Defense Strategies against Heterodera glycines
Source: Int J Mol Sci. 2023 Nov 12;24(22):16232. doi: 10.3390/ijms242216232 (PMC10671692; doi:10.3390/ijms242216232)
Supplement: Supplementary file 1 [file ijms-24-16232-s001.zip › Figure S1.pdf]

Figure S1- Amplifying the SCN 18S ribosomal gene from DNA extracted from the roots

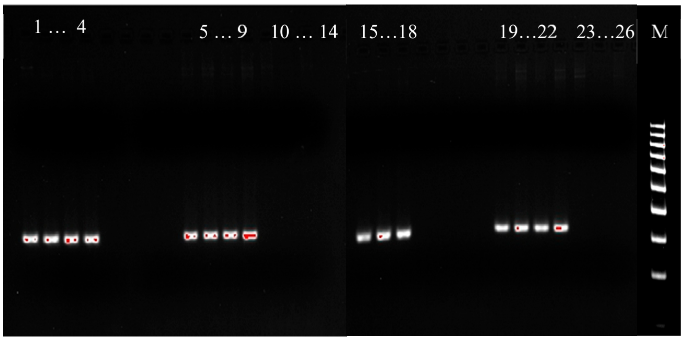

Figure S1. PCR amplification of 18S ribosomal RNA (250 bp band) in SCN, infected plants and Control at 5 and 10 dpi. Lanes 1 to 4 and Lane 15 to 18 are SCN. Lanes 5 to 9 are infected plants at 5 dpi. Lane 10-14 are Control plants at 5 dpi. Lane 19 to 22 are infected plants at 10 dpi. Lane 23 to 26 are control plants at 10 dpi.
