# Supplementary material for: Soybean–SCN Battle: Novel Insight into Soybean’s Defense Strategies against Heterodera glycines
Source: Int J Mol Sci. 2023 Nov 12;24(22):16232. doi: 10.3390/ijms242216232 (PMC10671692; doi:10.3390/ijms242216232)
Supplement: Supplementary file 1 [file ijms-24-16232-s001.zip › Table S11.pdf]

Table S11. Sets of primers and probes used for TaqMan assay to evaluate copy number variation (CNV) of *rhg1* and *Rhg4* genes

Gene	Primer sequences	Tm	Probe sequence	Tm
Rhg1	Fwd: GTTATTACTTCAATCGACGAGTGTGTTG	59	FAM: TCGGACACCTCAAAACT	68
	Rev: AAATATTTTCCAGTAAAATCAGATTAAAACTATACTTCA	58		
Lectin	Fwd: TCCCGAGTGGGTGAGGATAG	58	VIC: TGCTGCCACGGGAC	69
	Rev: CATGCGATTCCCCAGGTATG	59		
Rhg4	Fwd: GCTTGCAGACTGCCTTTGTTAAA	60	FAM: CAACAACTCGAAATTC	60
	Rev: TTTATAATTGATTTGCCAATCATATTGCACGA	65		
