# Supplementary material for: Soybean–SCN Battle: Novel Insight into Soybean’s Defense Strategies against Heterodera glycines
Source: Int J Mol Sci. 2023 Nov 12;24(22):16232. doi: 10.3390/ijms242216232 (PMC10671692; doi:10.3390/ijms242216232)
Supplement: Supplementary file 1 [file ijms-24-16232-s001.zip › Table S12.pdf]

Table S12. Primer sequences of nematode 18S ribosomal gene

Gene name	Primer sequences
SCN-18S-R	ACACGTCGCCGGTACAAGAC
SCN-18S-F	GCCATGCATGTGTAAGTTTAACCT
